# Epidemiology of confirmed measles virus cases, surveillance, incidence, and associated factors in Eritrea: 18-year retrospective analysis

**DOI:** 10.3389/fpubh.2023.1218317

**Published:** 2023-09-13

**Authors:** Samuel Tekle Mengistu, Oliver Okoth Achila, Asmerom Tesfagiorgis Tewelde, Mohammed Elfatih Hamida, Freweini Tekle, Issaias Michae, Mensura Said, Dawit Fsahatsion, Haimanot Abai, Rahel Mulugeta, Tsigehana Tsegai, Luwam Gilazghi Woldu, Wintana Yebio Werke

**Affiliations:** ^1^Nakfa Hospital, Ministry of Health Northern Red Sea Branch, Nakfa, Eritrea; ^2^Unit of Clinical Laboratory Sciences, Orotta College of Medicine and Health Sciences, Asmara, Eritrea; ^3^IDSR, CDC, Ministry of Health, Asmara, Eritrea; ^4^Unit of Microbiology, Orotta College of Medicine and Health Sciences, Asmara, Eritrea; ^5^Serology Department, National Health Laboratory, Asmara, Eritrea; ^6^Keren Hospital, Ministry of Health in Anseba Branch, Keren, Eritrea

**Keywords:** measles, surveillance, vaccination, epidemiology, spatial inequality

## Abstract

**Background:**

Despite the outstanding measles vaccine coverage (MVC) in Eritrea, sporadic outbreaks are not uncommon. Therefore, understanding the incidence of laboratory-confirmed measles virus cases, related factors, and spatial inequalities in testing and surveillance remains crucial. In this analysis, we evaluated the incidence and spatiotemporal distribution of measles in Eritrea. An evaluation of the factors associated with measles vaccination and IgM positive (+) febrile rash was also undertaken.

**Methods:**

A retrospective (period: 2002–2020) study was carried out by abstracting data from the integrated disease surveillance and response database (IDSR). Data was analyzed using descriptive statistics and binary logistic regression. Spatial variability and distribution of confirmed cases was evaluated using ArcGIS Pro version 3.0.1.

**Results:**

In total, 9,111 suspected cases, 2,767 [1,431 (51.7%) females] were serologically tested. The median (IQR) age, minimum-maximum age were 7 years (IQR: 4–14 years) and 1 month-97 years, respectively. Among the 608(21.9%) laboratory-confirmed cases, 534 (87.8%) were unvaccinated and 53 (9.92%) were < 1 year old. The crude incidence rate for MV was 14/100,000 persons. The age-specific positivity rate per 100,000 suspected cases tested was 21.5 with individuals >30 years presenting with the highest rates (69.9/100,000). Higher odds (OR) of MV test positivity was associated with age at onset – higher in the following age-bands [10–14 years: OR = 1.6 (95%CI, 1.1–2.2, value of *p* = 0.005); 15–29 years: OR = 7.0 (95%CI, 5.3–9.2, value of *p* = 0.005); and > =30 years: OR = 16.7 (95%CI, 11.7–24) *p* < 0.001]. Other associations included: Address – higher in Anseba (OR = 2.3, 95%CI: 1.7–3.1, value of *p*<0.001); Debub (OR = 2.7, 95%CI: 1.9–3.9, value of *p* < 0.001); Gash-Barka (OR = 15.4, 95%CI: 10.9–21.7, value of *p* < 0.001); Northern Red Sea (OR = 11.8, 95%CI: 8.5–16.2, value of *p* < 0.001); and Southern Red Sea (OR = 14.4, 95%CI: 8.2–25.2, value of *p* < 0.001). Further, test positivity was higher in health centers (OR = 2.5, 95%CI: 1.9–3.4, value of *p* < 0.001) and hospitals (OR = 6.8, 95%CI: 5.1–9.1, value of *p* < 0.001). Additional factors included vaccination status - higher in the unvaccinated (OR = 14.7, 95%CI: 11.4–19.1, value of *p* < 0.001); and year of onset of rash – (higher >2015: OR = 1.4, 95%CI: 1.1–1.7, value of *p*<0.001). Uptake of measles vaccine associated with a similar complement of factors.

**Conclusion:**

In large part, efforts to eliminate measles in Eritrea are hindered by disparities in vaccine coverage, under-surveillance, and low vaccination rates in neighboring countries. Enhanced surveillance and regional micro planning targeting hard-to-reach areas can be an effective strategy to improve measles elimination efforts in Eritrea.

## Introduction

Measles virus (MV), a member of the genus *Morbilivirus*, family *Paramyxoviridae*, is an antigenically monotypic, enveloped, single-stranded (SS) virus with a non-segmented negative (−) sense RNA genome (1). Like other *Morbiliviruses*, MV is highly contagious [basic reproductive number (*R*_0_ = 10–18) and can cause multiple complications (1)]. Following exposure *by* respiratory route or contact with respiratory droplets, up to 90% of susceptible people can develop systemic infection within 10–14 days (up to 3 weeks in adults) (2). The 1 to 3 day prodromal phase is hallmarked by fever, malaise, and the three C (cough, coryza, and conjunctivitis) (3). A common finding at this stage is Koplik’s spot and an erythematous maculopapular rash. Although MV infection is self-limiting, common sequelae include transient immunosuppression, diarrhea, otitis media, laryngotracheobrochitis, pneumonia (accounts for 90% of measles mortality), and post-viral encephalitis. On rare occasions, it can lead to subacute sclerosing panencephalitis, a potentially fatal central nervous system (CNS) complications (1).

Before the introduction of the measles vaccine in the 1960s, MV epidemics occurred periodically (every 2–3 years), with approximately 2.6 million mortalities annually (3). However, the scale-up of vaccine coverage, particularly in low- and middle-income countries (LMICs) in the 1990s triggered a significant decline in incidence, morbidity, and mortalities (4). Globally, data shows that from 1990 to 2019; there was a decline from 80 933,448.62 [Age standardized rate (ASR): 1278.81 per 100,000] to 12,806,077.45 (ASR: 191.04 per 100,000) in MV cases translating into an annualized ASR decrease of 6.13% (95% CI 5.41–6.84%) (4). In terms of the 5 sociodemographic index (SDI) sub-regions, low SDI and low-middle SDI regions had higher ASR in 2019, whereas lower ASR was observed in high SDI sub regions [11.59 per 1,00,000 in high SDI vs. 307.32 per 1,00,000 in low SDI region (4)]. The data from global burden of disease (GD) database is corroborated by measles case-based surveillance data in Africa. According to this source, the number of cases in Africa decreased from 520,102 cases in 2000 to 98,621 cases in 2015 translating into a corresponding incidence decline of 841 per 1,000,000 to 100 per 1,000,000 in the same period (3). The report also concluded that measles vaccination [measles-containing vaccine 1 (MCV1), measles-containing vaccine 2 (MCV 2) and supplemental immunization activities (SIAs)] prevented 23.2 million deaths from 2000 to 2018 (3). Importantly, declines in mortality have paralleled steep declines in MV-associated disability-adjusted life-years (DALYs). From 1990 to 2019 for instance, the DALY rates decreased from 2.7 (Uncertainty interval (UI):0.9–5.6) to 0.3 (UI: 0.1–0.6), translating into an estimated percentage change (EAPC) of −89.8%(−92.3–86.8%) (5). However, measles still account for a significant number of deaths globally (>140,000 persons in 2018, mostly children <5 years) (6).

By most accounts, the remarkable decline in the burden of measles in Sub-Saharan Africa (SSA) is closely intertwined with multiple policy prescriptions of the World Health Organisation (WHO) policy prescription on immunization. In 2008, following the earlier standards set by the 2006–2015 Global Immunisation Vision and Strategy; the WHO adopted its four-point pre-elimination plan for the African Region (AFR) (7). These included: (1) >98% reduction in measles mortality with the year 2000 as the baseline, (2) annual incidence of measles incidence <5 per 1,000,000 cases nationally, (3) >90% MCV1 coverage nationally >80% coverage in districts, and (4) >95% SIA in all districts. Specific surveillance performance objectives were also embedded in the document. In 2011, these goals were adopted by the WHO AFR 2020 measles elimination plan, and subsequently incorporated into its regional immunization strategic plan (7).

In Eritrea, the Expanded Programme on Immunisation (EPI) began in 1980 (pre-independence era). The original EPI included the provision of the first dose of measles-containing vaccine (MCV1) at 9 months of age. However, the coverage of MCV1 in this period was very low, 34% (8). In 2003, the country implemented SIAs for the first time for the age group 9 months to 14 years. This was followed by the implementation of case-based surveillance in 2005, and MCV2 in 2012. According to the WHO-AFR goals, and its second national Strategic Development Plan (HSSDP-II) (2017–2021); Eritrea adopted the >95% (MCV1)-90% (MVC2) as a principle part of its national multiyear plan for immunization. Currently, the WHO and United Nations Children’s Fund (WHO/UNICEF) score card on vaccination estimates suggest that Eritrea has consistently reported >95% MCV1 since 2003 and > 88% MCV2 coverage since 2015 (8).

Although the adoption of these policies was broadly successful; The MCV2 and SIAs targets, surveillance performance targets, and incidence rates <5 per 1,000,000 cases nationwide were not met (9). In other settings across WHO-AFR regions, underperformance in these indicators has been associated with poor access to community-linked immunization services; inadequate infrastructure for laboratory-supported case-based surveillance, poor quality of data, and reduction in vaccine effectiveness, among others (9). Importantly, others have opined that national WHO-UNICEF estimates (vaccination or measles burden) can mask within-country disparities, and context-specific social and economic drivers of measles infection or under-vaccination. For this reason, clusters of unvaccinated or under-vaccinated individuals are known to exist even in countries with relatively high MCV coverage like Eritrea. This problem, it can be argued, can be addressed by renewed research effort, particularly in hard-to-reach communities. Unfortunately, only a limited number of investigations have focused on spatiotemporal heterogeneity in measles incidence, testing and MCV coverage. Data on demographic correlates of measles infection at the local level is also lacking. Much of this description applies to Eritrea. In this analysis, our objective was to evaluate the incidence and spatiotemporal distribution of measles in Eritrea. This study sought to evaluate the incidence and spatiotemporal distribution of measles in Eritrea. Furthermore, an evaluation of the factors associated with measles vaccination and IgM positive (+) febrile rash was attempted.

## Methods

### Study design, population, and settings

We conducted a retrospective analysis of data collected in Eritrea from 2002 to 2020. Eritrea is a country located in the upper part of the Horn of Africa. The country is bounded to the east by the Red Sea; to the North and west by Sudan; to the South by Ethiopia and to the South East by Djibouti. It has a surface area of ~124,000 Km^2^ and a total population of 3,905,066, including ~117,152 surviving infants as of 2022. According to the Inter-Agency Group of the United Nations for Child Mortality Estimation, the mortality rate, and infant mortality rate in 2020 were 38 per 1,000 live births and 18 per 1,000 live births, respectively (10). Administratively, the country is divided into six *Zobas* (regions): Maekel (central region), Gash Barka, Anseba, Debub, Debubawi Keih Bahri (DKB) (Southern Red Sea) and Semanawi Keih Bahri (SKB) (Northern Red Sea). The regions are further subdivided into 58 sub-zones, 699 administrative areas, and 2,564 villages. Healthcare is provided, almost exclusively, by the Eritrea Ministry of Health through a three-tier system, namely primary care level (serves ~500–2000 people), secondary care level (serves ~5,000–10,000) and tertiary care level (serves ~50,000–100,000). Using the reaching every district approach, the country’s EPI program, which is housed as a unit within the Department of Public Health; delivers immunization services, 6 days per week, through 302 (85%) health facilities and 450 outreach sites (8). In addition, country implements Periodic Intensified Routine Immunization (PIRI) services, every quarter for nomadic and semi-nomadic population groups and people living in 16 hard-to-reach districts. As of December 2021, the national immunization target includes antigens against 12 vaccine preventable diseases. Overall, data from vaccination cards and mother’s reports suggest that in 2020, 99.4, 98.9, 98.8, and 96.9% received required doses of Bacillus Calmette Guérin (BCG) vaccine, oral polio vaccine (OPV), PENTA-3 (vaccine for Hepatitis B virus (HBV), Diptheria-Pertussis-Tetanus (DPT), Haemophilus influenza type b) and measles-mumps-rubella (MMR) - MCV (MCV1 at 9 months and MCV2 at 15 months), respectively.

### Data source and study variables

In Eritrea, the national serological laboratory conducts measles case-based surveillance to confirm measles cases is undertaken by the national serological laboratory. The surveillance protocols used by the measles serological laboratory are based on WHO-AFR standards (11). It includes individual case investigation and blood specimen collection for laboratory testing (9) in national or regional reference laboratories. At present, molecular techniques [real-time polymerase chain reaction (RT-PCR) and sequence analysis] or cell culture are not available in the national laboratory. In general, the data used in this analysis were obtained from the integrated disease surveillance and response database (IDSR). The database contains information on the number of measles tests conducted per year at the National Hospital Laboratory (the only testing center in Eritrea). The data used for age standardization were obtained from the Eritrea Statistics Office. The variables considered in this analysis included total notified cases, number of notified cases which were tested, positive immunoglobulin M (IgM) (+), Ig M−/ Intermediate, and epidemiological linked cases (EPI-linked), and final outcome of MCV infection. Additional information included patient sex, age at onset of rash, address, health facility, vaccination status, last vaccination, and date of the onset of rash.

### Data collection procedures and data quality

In this study, data (spanning the period from 2002 to 2020) was abstracted IDSR using a form designed for this purpose. The variables captured included age at the beginning of the rash, years, address (region / zone), facility level, vaccination status, and last vaccination. To ensure compliance with specific ethical requirements, all participant data were de-identified. At this stage, the data were managed using CSPro software (version 7.0).

### Operational definitions

■ A suspected measles case was defined according to the WHO-AFR Measles and Rubella Surveillance Guideline [WHO-AFR] – persons with a generalized maculopapular rash, fever, plus one or more of the following: coryza, cough, conjunctivitis, OR any person in whom a clinician suspects measles (12).
■ A confirmed measles case was defined as the presence of measles-specific IgM antibodies (IgM^+^) of a titer that exceeded the thresholds indicative of infection in people without evidence of vaccination in the last 30 days (13).
■ Epidemiologically linked case refers to a suspected case that has been linked (in person, place, and time) to a laboratory confirmed case (13).
■ A non-measles case was defined as any notified case/suspected case that was measles-specific IgM (−) after testing as per established protocols.
■ The status of measles vaccination was categorized into vaccination (at least MCV1), non-vaccination, and unknown status.

### Main results of the study

The outcome variables included the crude incidence rate (CIR), age adjusted incidence rates, and age-specific measles IgM positivity. Additional outcomes included factors associated with measles-specific IgM positive febrile rash and vaccination. The spatial distribution of measles tests and positive cases from 2002 to 2020 across Eritrean sub-zones was also evaluated.

### Statistical analyses

Data was analyzed using IBM® SPSS® Statistics for Windows, version 26 (IBM Corp., Armonk, N.Y., USA). Crude incidence rates (CIR), age standardized rates (ASR), and specific measures of central tendency, medians [±Interquartile ranges (IQR)] and means [±standard deviations (SD) were computed where applicable]. The CIR was calculated by dividing the total number of confirmed cases (by laboratory serology) per year or age category as the number of the total population. The numbers used in these denominators were obtained from the Eritrea National Statistics Office (14). To test for differences in medians or frequencies between groups, Kruskal-Wallis, Mann–Whitney *U*, or Chi-squire statistics (2) were used. Subsequently, binary logistic regression models were fitted to identify the predictors of measles test positivity or vaccine uptake of vaccine in the population. The results were finally presented in tables, graphs, and figures. Furthermore, the spatial variability in the tests and the distribution of confirmed cases during the study period was calculated using ArcGIS version 10.1.

### Ethical considerations

The study was carried out according to the Declaration of Helsinki and was approved by Ethical Committee of the Eritrean Ministry of Health (EMH). Written consent was not obtained from the patients. Instead, the data were identified and all ethical and professional considerations undergirding confidentiality/or others protections of patient information were rigorously implemented.

## Results

From 2002 to 2020, ~ 9,111 cases of febrile rash notifications were reported in Eritrea. However, of 2,767 (30.4%) cases were tested; MV IgM was 607 (21.9%) and 82 (3% of the tested) were epidemiologically linked. Mean (±SD), 10.5 months (±10.4 months) with a minimum age – maximum age of the cases was 1 and 97 months, respectively. The overall CIR of MV was 14 per 100,000. Overall, 17 (3% of the total measles) cases died due to measles and/or its complications. This translates into a reported MV-related death rate of 24.6 per 1,000. See Table 1.

**Table 1:** Distribution of measles serologic test results across years from 2002 to 2020 in Eritrea.

Year	Total cases notified	Tested *n* (%)	IgM+	Ig M−/Intermediate	Epi link	Crude rate per 100,000	Measles death	MDR/1000
2002	85	79 (93)	23	56	0	0.8841	0	0
2003	363	99 (27)	48	51	21	1.7969	1	14.5
2004	48	40 (83)	9	31	8	0.3417	0	0
2005	95	89 (94)	14	75	6	0.5166	0	0
2006	134	128(96)	4	125	0	0.1076	4	1,000
2007	58	58(100)	0	58	0	0.03486	0	0
2008	65	64(98)	0	64	0	0.03389	0	0
2009	73	52(71)	3	49	0	0.0988	0	0
2010	171	171(100)	5	166	0	0.1601	0	0
2011	147	147(100)	14	133	0	0.4357	0	0
2012	682	305(45)	119	186	40	3.6	1	6.2
2013	621	150(24)	47	103	0	1.382	4	85
2014	127	114(90)	1	113	7	0.0285	0	0
2015	468	202(43)	91	111	0	2.528	6	65.9
2016	726	364(50)	79	285	0	2.1378	0	0
2017	3,150	196(6.2)	60	136	0	1.5794	1	16.6
2018	1,023	176(17)	70	106	0	1.7925	0	0
2019	545	191(35)	8	183	0	0.1993	0	0
2020	620	142(23)	13	129	0	0.3151	0	0
Total	9,111	2,767(30.4)	607	2,160	82	14	17	24.6

Attack rates; Number of measles/total number of patients tested. MDR; measles death rate.

### Crude incidence rate, age adjusted incidence rates, and age-specific measles IgM positivity

In this analysis, a higher CIR and age-specific incidence of measles was observed in adults: = < 4 years, 11.7 per 100 samples tested; 5–9 years, 8.6 per 100 samples tested; 9–14 years, 17.4 per 100 samples tested; 15–29 years, 49.8 per 100 tested; > = 30 years, 69.9 per 100 tested. The CIR (ASR) during the study period: = < 4 years, 3 per 100,000 (14.5 per 100,000); 5–9 years, 2.5 per 100,000 (15.34 per 100,000); 9–14 years, 2 per 100,000 (14.6 per 100,000); 15–29 years, 6.4 per 100,000(43.65 per 100,000); > = 30 years, 4.2 per 100,000 (76.79 per 100,000). Furthermore, there was a pan age group increase in measles incidents from 2015 to 2018. See Table 2.

**Table 2:** Crude incidence rate per 100,000, age adjusted incidence rates (ASR) per 1,000,000 and age specific positivity among 100 individuals the tested for measles in Eritrea, 2002–2020.

CIR (ASR)						Age specific positivity rate per 100 tested					
Year	≤4	5–9	9–14	15–29	≥30	≤4	5–9	9–14	15–29	≥30	Total
2002	0.2 (0.78)	0.2(0.77)	0.04(0.76)	0.36(2.18)	0.08(4.36)	25	15.6	100	90	66.7	33.3
2003	0.12(0.59)	0.47(1.5)	0.63(1.5)	0.59(4.42)	–	30	70.6	69.6	83.3	–	66.7
2004	0.04(0.3)	0.11(0.3)	0.08(0.29)	0.08(0.84)	0.04(1.68)	7.7	18.8	33.3	100	100	23.7
2005	0.07(0.46)	0.22(0.45)	–	0.15(1.27)	0.07(2.54)	7.4	15.4	–	66.7	66.7	15.7
2006	–	0.07(0.09)	0.04(0.09)	–	–	–	3.4	4.2	–	–	2.3
2007	–	–	–	–	–	–	–	–	–	–	–
2008	–	–	–	–	–	–	–	–	–	–	–
2009	–	0.03(0.09)	–	0.03(0.24)	0.03(0.49)	–	3.7	–	50	100	5.8
2010	–	–	0.03(0.14)	0.1(0.39)	0.03(0.79)	–	–	6.7	33.3	100	2.9
2011	0.03(0.39)	0.09(0.38)	0.03(0.37)	0.19(1)	0.09(2.15)	2.4	5.2	4.5	31.6	50	9.5
2012	0.27(3.19)	0.12(3.13)	0.06(3.1)	2(8.86)	1.15(17.7)	12.7	7.1	9.5	61.7	78	39.3
2013	0.06(1.22)	0.06(1.2)	0.15(1.19)	0.76(3.4)	0.35(6.81)	5.4	5.4	33.3	57.8	84.6	31.3
2014	0.03(0.03)	–	–	–	–	2	–	–	–	–	–
2015	0.14(2.24)	0.28(2.2)	0.17(2.17)	0.94(6.22)	1(12.45)	16.7	20.4	25	61.8	83.7	45.3
2016	0.35(1.89)	0.38(1.86)	0.08(1.84)	0.46(5.26)	0.87(10.5)	12.9	10.5	7.3	35.4	78	21.7
2017	0.63(1.4)	0.21(1.37)	0.37(1.36)	0.21(3.89)	0.13(7.78)	36.4	15.4	29.8	38.1	55.6	30.3
2018	0.67(1.59)	0.18(1.56)	0.26(1.54)	0.41(4.41)	0.26(8.83)	44.1	22.6	35.7	43.2	50	39.4
2019	0.1(0.18)	0.02(0.17)	–	0.05(0.49)	–	4.4	1.6	–	18.2	–	3.7
2020	0.1(0.28)	0.02(0.27)	0.02(0.27)	0.07(0.78)	0.07(1.55)	5.8	2	5	37.5	42.9	7.8
Total	3(14.54)	2.5 (15.34)	2 (14.6)	6.4 (43.65)	4.2 (77.69)	11.7	8.6	17.4	49.8	69.9	21.5

Attack rates_age specified_, number of measles cases/number of people tested positive in that age group.

### Factors associated with measles IgM positive febrile rash

Among the measles positive individuals’ females account for 55% (*n* = 334). The median age of positive individuals is higher than that of negative individuals (18 [7–29] vs. 6 [3–10] years, Kruskal–Wallis test, *p* < 0.001). The highest proportion of positives lie in the age group 15–29 years: 212 (35.1%) followed by more than 30 years 146 (24.2%), less than 4 years 102 (16.9%), 5–9 years 79 (13.1%), and 10–14 years 65 (10.8%), chi-square test *p* < 0.001. Among the countries zones, a higher positivity was observed in the regions of NRS 145 (23.8%) and Anseba 142 (23.4%), chi-square *p* < 0.001. No apparent differences in the number of incidence cases by sex were observed (Table 1). Higher odds of MCV positivity were observed in Gash-Barka [OR = 15.4(10.9–21.7), *p* < 0.001], Southern Red Sea [OR = 14.4 (8.2–25.2), *p* < 0.001], Northern Red Sea [OR = 11.8 (8.5–16.2), *p* < 0.001], Debub [OR = 2.7 (1.9–3.9), *p* < 0.001] and Anseba [OR = 2.3 (1.7–3.1), *p* < 0.001] when compared to Maekel region. Hospitals’ samples showed higher of odds of MCV positivity (224, 45.3%) when compared to health centers or stations (OR = 6.8, *p* < 0.001, hospital vs. health station). Higher odds of test positivity were also observed in unvaccinated individuals [OR = 14.7 (11.4–19.1), *p* < 0.001]. See Table 3.

**Table 3:** Factors associated with measles status in Eritrea, 2002–2020.

Characteristics	Measles status			Value of *p* (χ^2^)	OR (95%CI)	Value of *p*
	Positive (%)	Negative (%)	Intermediate *N* (%)			
**Gender**						
Male	267 (44.4)	990 (48.3)	50 (56.3)	0.06 (5.5)	1(*Ref.*)	
Female	334 (55.6)	1,058 (51.7)	38 (43.7)		1.18 (0.9–1.42)	0.06
Age at onset of rash, median (IQR)	18 (7–29)	6 (3–10)	11 (5–18)	**<0.001** ^**a**^		
= < 4	102(16.9)	731 (35.4)	18 (20.7)	**<0.001 (645.7)**	1(*Ref.*)	
5–9	79 (13.1)	805 (39)	18 (20.7)		0.7 (0.5–0.9)	**0.02**
10–14	65 (10.8)	274 (13.3)	21 (24.1)		1.6 (1.1–2.2)	**0.005**
15–29	212 (35.1)	203 (9.8)	19 (21.8)		7 (5.3–9.2)	**<0.001**
> = 30	146 (24.2)	53 (2.6)	11 (12.6)		16.7 (11.7–24)	**<0.001**
**Address**						
Maekel	91 (15)	962 (46.4)	33 (37.5)	**<0.001 (501.2)**	1(*Ref.*)	
Anseba	142 (23.4)	637 (30.8)	24 (27.3)		2.3 (1.7–3.1)	**<0.001**
Debub	64 (10.5)	239 (11.5)	13 (14.8)		2.7 (1.9–3.9)	**<0.001**
Gash-Barka	133 (21.9)	90 (4.3)	4 (4.5)		15.4 (10.9–21.7)	**<0.001**
NRS	145 (23.8)	120 (5.8)	13 (14.8)		11.8 (8.5–16.2)	**<0.001**
SRS	33 (5.4)	24 (1.2)	1 (1.1)		14.4 (8.2–25.2)	**<0.001**
**Health facility**						
Health station	90 (18.2)	624 (47.2)	22 (37.3)	**<0.001 (198)**	1(*Ref.*)	
Health center	180 (36.4)	478 (36.1)	23 (39)		2.5 (1.9–3.4)	**<0.001**
Hospital	224 (45.3)	221 (16.7)	14 (23.7)		6.8 (5.1–9.1)	**<0.001**
**Vaccination status**						
At least one dose	74 (12.2)	1,380 (66.6)	46 (52.3)	**<0.001** **(604.7)**	1(*Ref.*)	
Unvaccinated	534 (87.8)	654 (31.6)	42 (47.7)		14.7 (11.4–19.1)	**<0.001**
Unknown	–	38 (1.8)	–			
Last vaccination	2009 (2004–2014)	2011 (2004–2015)	2006 (2003–2013)			
≤2009	34 (45.9)	744 (54)	31 (67.4)	0.07 (5.3)	1(*Ref.*)	
≥2010	40 (54.1)	635 (46)	15 (32.6)		1.4 (0.8–2.2)	0.15
Year of onset of rash	2012 (2010–2017)	2015 (2012–2016)	2016 (2007–2018)	0.07^a^		
≤2014	287 (75.1)	1,175 (91.9)	36 (82.9)	**<0.001** **(72)**	1(*Ref.*)	
≥2015	91 (24.1)	104 (8.1)	7 (17.1)		1.4 (1.1–1.7)	**<0.001**

NRS, Northern Red Sea; SRS, Southern Red Sea; IQR, Interquartile Range. The bold values indicated the significance; ^a^independent samples *t*-test.

### Factors associated with measles vaccination

In the bivariate analysis, we demonstrated that unvaccinated individuals were older with a median age (IQR) of 9 (IQR: 1–25) years vs. the vaccinated 3 (IQR: 3–8) years, Mann–Whitney *U* test *p* < 0.001. Additionally, vaccination uptake was associated with age at the time of rash appearance, address, type of health facility, and year of onset of rash. Consequently, 276 (22.5%), 235 (19.26%), 150 (12.3%), 365 (29.8%) and 198(97.1%) of patients = <4 years, 5–9 years, 10–14 years, 15–29 years and > =30 years were not vaccinated. In the respective administrative zones, namely: Maekel, Anseba, Debub, Gash-Barka, NRS, and SRS; 270 (25.62%), 332 (41.4%), 153 (48.57%), 189 (84.00%), 232 (84.06%), 55 (94.83%) of patients presenting morbiliform rash were unvaccinated. Additionally, 228 (30.9%), 304 (44.7%) and 315 (66.63%) of patients with rashes in health stations, health centers and hospitals were not vaccinated. Last but not least, = < 2014 vs. > =2015 analysis of = 2014 vs. > = 2015 of the vaccination status of individuals who presented a rash indicated that 720 (49.38%) and 510 (40.13%) were not vaccinated.

In the multivariate analysis, a high odd of being unvaccinated was associated with the age at onset of rash [1.11 (95% CI 1.10–1.13), *p-*value<0.001]; administrative zone [SRS: aOR = 23(95% CI: 4.6–114), *p-*value<0.001]; [NRS: aOR = 7.6 (95% CI: 4.6–12.7)]; [Anseba: aOR = 2.4 (95% CI: 1.8–3.2)] [Debub: aOR = 1.8 (95% CI, 1.2–2.6)]; and health facility [Health centre: 0.95 (95% CI, 0.72–1.3)]; [hospital: 5.1 (955 CI, 3.6–7.2), *p-*value<0.001]. Lastly, there was a notable association between year of rash onset and vaccination status [0.94 (95% CI, 0.90–0.99), value of *p* = 0.02]. In other words, for each increase in year, there was a 6% increase in vaccination status of individuals with febrile rash (*p* = 0.02) (Table 4).

**Table 4:** Factors associated with measles vaccination among individuals with rash in Eritrea, 2002–2020.

Characteristics	Vaccination status		*value of p* (*χ*2)	OR (95%CI)	*value of p*	aOR (95%CI)	value of p
	At least once (%)	Unvaccinated (%)					
**Gender**							
Male	701 (54.6)	583 (45.4)	0.8 (0.06)	1(*Ref.*)			
Female	780 (55.1)	636 (44.9)		0.9 (0.8–1.1)	0.7		
Age at onset of rash	**3 (3–8)**	**9 (1–25)**	**<0.001** ^a^			1.11 (1.10–1.13)	**<0.001**
= < 4	563 (67.1)	276 (22.5)	**<0.001 (686.5)**	1(*Ref.*)			
5–9	661 (44.20)	235 (19.2)		0.7 (0.5–0.8)	**0.002**		
10–14	206 (13.8)	150 (12.3)		1.4 (1.1–1.9)	**0.002**		
15–29	61 (4.1)	365 (29.8)		12.2 (8.9–16.5)	**<0.001**		
> = 30	6 (2.9)	198 (97.1)		67.3 (29.5–153.5)	**<0.001**		
**Address**							
Maekel	784 (74.38)	270 (25.62)	**<0.001 (532.2)**	1(*Ref.*)		1(*Ref.*)	
Anseba	470 (58.6)	332 (41.4)		2 (1.6–2.4)	**<0.001**	2.4 (1.8–3.2)	**<0.001**
Debub	162 (51.43)	153 (48.57)		2.7 (2.1–3.5)	**<0.001**	1.8 (1.2–2.6)	**<0.001**
Gash-Barka	36 (16.0)	189 (84.00)		15.2 (10.4–22.3)	**<0.001**	10.9 (6.1–19.6)	**<0.001**
NRS	44 (15.94)	232 (84.06)		15.3 (10.7–21.7)	**<0.001**	7.6 (4.6–12.7)	**<0.001**
SRS	3 (5.17)	55 (94.83)		53.2 (16.5–171.5)	**<0.001**	23 (4.6–114)	**<0.001**
**Health facility**							
Health station	509 (69.1)	228 (30.9)	**<0.001 (162.3)**	1(*Ref.*)		1(*Ref.*)	
Health center	337 (52.6)	304 (47.4)		1.8 (1.4–2.2)	**<0.001**	0.95 (0.72–1.3)	0.7
Hospital	144 (31.37)	315 (68.63)		4.8 (3.7–6.2)	**<0.001**	5.1 (3.6–7.2)	**<0.001**
Year of onset of rash	2015 (2010–2017)	2013 (2010–2016)	**<0.001** ^**a**^			0.94 (0.90–0.99)	**0.02**
≤2014	738 (50.62)	720 (49.38)	**<0.001 (15.8)**	1(*Ref.*)			
≥2015	761 (59.87)	510 (40.13)		0.6 (0.5–0.8)	**<0.001**		

NRS, Northern Red Sea; SRS, Southern Red Sea; IQR, Interquartile Range. The bold values indicated the significance; ^a^independent samples *t*-test.

### Spatial distribution of measles tests and positive cases from 2002 to 2020 across Eritrean sub-zones

**Figure 1:**
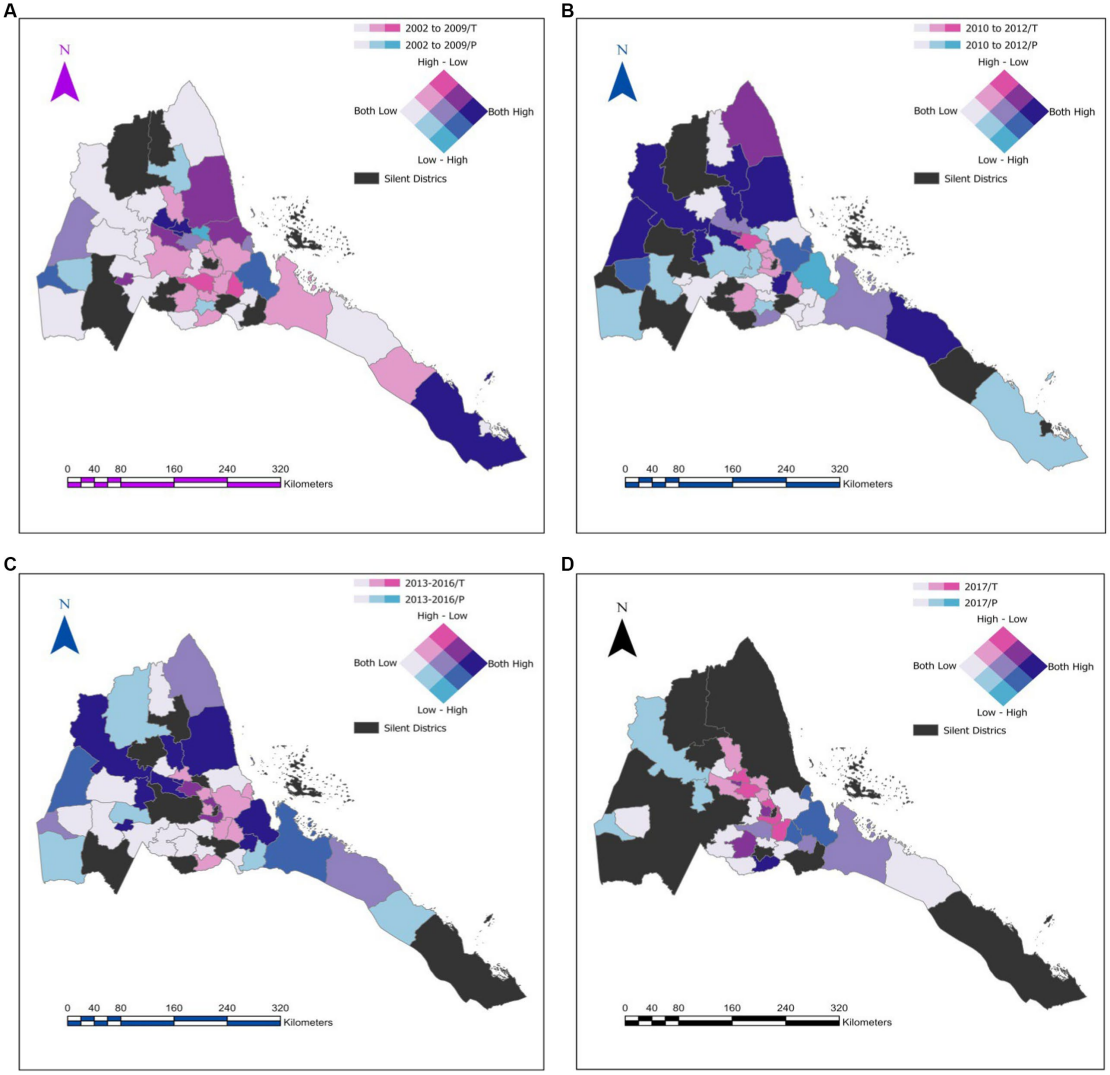
Geospatial symbology for the distribution of measles by quantiles of numbers of serologic tests (T), and positive (P) results across the country. **(A)** Represents for the year band of 2002–2009. **(B)** Represent 2010–2012. **(C)** Represent 2013–2016 and **(D)** is for 2017–2020.

Figures 1A–D presents the distribution of measles by quantiles of the number of serologic tests (T) and positive (P) results in Eritrea from 2002–2020. On the main, the figures demonstrate that measles testing is highly heterogeneous and is mostly concentrated in subzones around Maekel (central administrative district) where the testing laboratory is located. Furthermore, between 2017 and 2020, testing in some of the most vulnerable regions was low.

## Discussion

In an era where measles elimination is increasingly becoming a reality even in countries in some of the most disadvantaged parts of the world; sound evidence on incidence, trends, and associated factors at the national and rural level is essential. The current exploratory study aims to investigate these themes in Eritrea. In each and every one of these domains, we discovered multiple standout features. Foremost is the fact that the incidence of measles in Eritrea undulated between moderate to low rate periods over the course of 18 years. Highest CIR > 1 per 100,000 were reported in 2003 (1.79 per 100,000); 2012 (3.6 per 100,000); 2013 (1.38 per 100,000); 2015 (2.52 per 100,000); 2016 (2.14 per 100,000); 2016 (2.13 per 100,000); 2017 (1.58 per 100,000); and 2018 (1.79 per 100,000). These results are in line with data from a recent study on progress towards measles elimination in Eritrea: 2003–2018 (8). Importantly, estimates are relatively lower than what has been reported in the African region (567 per 1 million) (15).

In a recent work modeling the so-called canonical path to measles elimination, Graham and colleagues argued that in most countries; the path to elimination is tripartite. It begins with regime of uncontrolled endemic transmission, characterized by a high incidence of measles and low year-to-year variability. This progresses to a second phase characterized by low incidence but high variability. The final phase is that of the elimination state, i.e., zero incidence and zero variability (16). Based on our data, we can conclude that Eritrea is in the second stage of the canonical path. According to our analysis, Eritrea has typical second phase patterns characterized by a relatively low incidence of MCV and a high year-to-year variability. In a separate analysis, we demonstrate that Eritrea has lingered in this stage for a relatively long time.

Although the present analysis provides a lot of insight on the state of measles elimination efforts in Eritrea, the problem of data reliability should be acknowledged. Indeed, the likelihood that this study has underestimated the incidence of measles in the country is hard to discount. Unlike the Yahdego et al. study which estimated measles incidence based on cases confirmed by laboratory, epidemiological linkage, and clinical criteria (i.e., as if the country is in the pre-elimination phase); our study relied entirely on laboratory confirmed cases. Adoption of this approach was based on the fact that clinical case definition has a limited role in measles case classification in some pre-elimination settings. In other words, the likelihood that measles-like rash symptoms are due to other diseases like Scarlet fever, Rubella, Epstein–Barr virus, Parvovirus B19 Infection (Fifth disease), Chikungunya, and Dengue virus (which are increasingly affecting these populations) is high in some settings. Although outbreaks of Dengue virus, Rubella, and Chikungunya have been reported in Neighboring countries, data from Eritrea is limited. Therefore, the contribution to notified cases is hard to discern.

Additionally, we are aware that the exclusive use of laboratory confirmed cases in estimating the incidence of measles incidence in recent years is severely undermined by suboptimal tests of suspected cases. With the exception of 2003, the frequency of serological tests was higher (Minimum 71.23% – Maximum, 100%) prior to 2012; a sharp decline was observed in the subsequent years. In 2017 for instance, the testing frequency of suspected cases reached a nadir of 6.22%. Taking all this into account (even the possibility that a substantial number of suspected cases were due to other causes), the possibility that our study has underestimated the true incidence of measles is substantial. The underreporting of measles can also be attributed to existing surveillance methods. In general, measles surveillance in WHO-AFR is largely based on passive surveillance (based on hospital admissions or contact). In areas where access to hospital or other care facilities is compromised by sub-optimal awareness, poor health seeking behavior, long distances to facilities, vaccine hesitancy, self-medication, and use of alternative medicine; under-reporting of measles cases is inevitable. As such, reported cases reflect a small proportion of the actual case burden in most countries in WHO-AFR (17). Regarding vaccine hesitancy, an issue that has emerged as a major threat to vaccine coverage in recent years, we will note that the problem is under-studied in Eritrea and should be a prominent research theme in future investigations.

**Figure 2:**
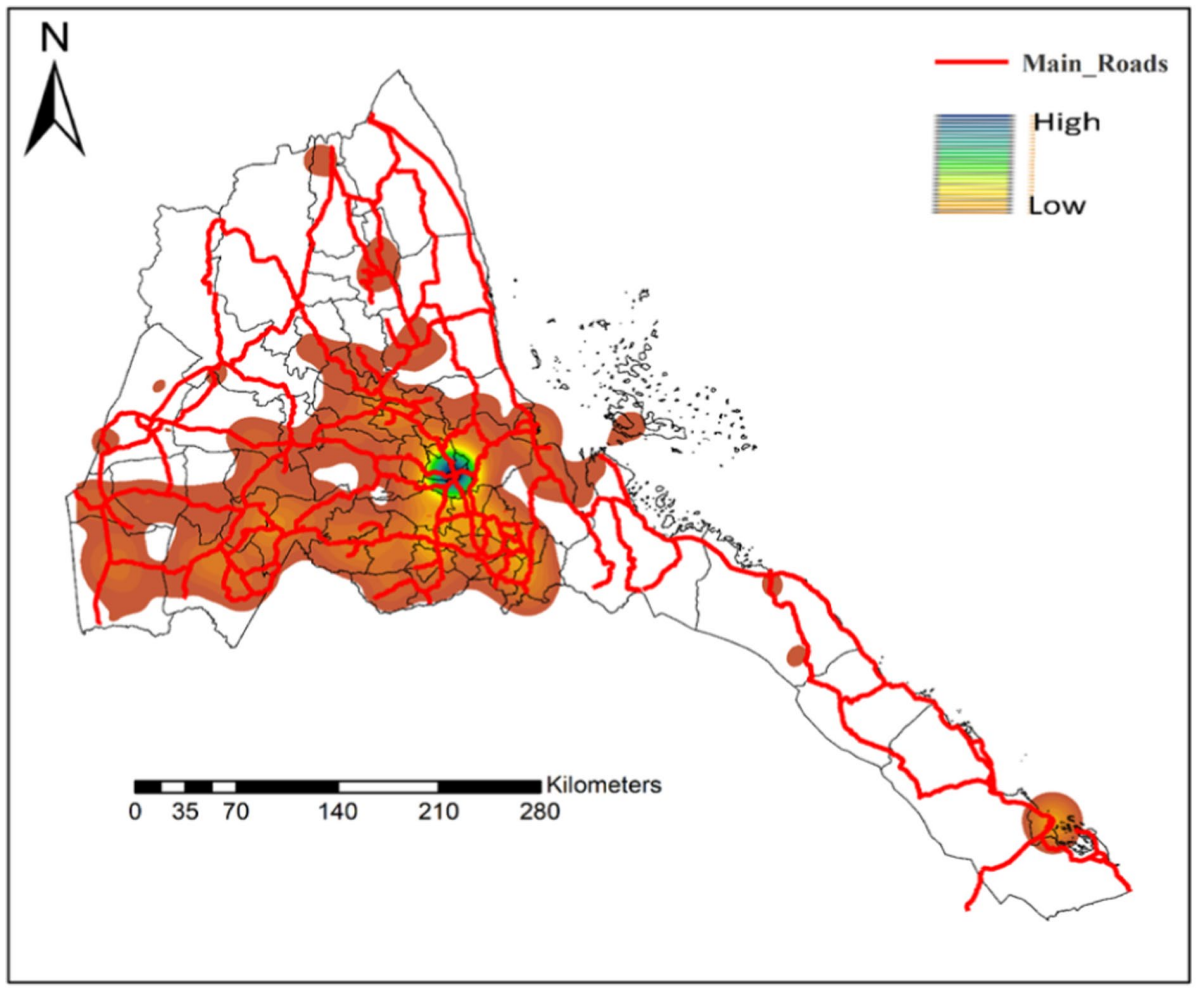
Distribution of health facilities across Eritrea presented by Kernel density. The red lines represent the main road connecting the different parts of the country.

In recent years, potential underreporting of measles cases has been investigated by multiple authors with remarkable results. For example, in the a study conducted in Oromia region in Ethiopia, Poletti et al. reported that the effectiveness of passive surveillance based on hospital admissions or contact decreases dramatically with travel distances from the hospital, becoming negligible beyond 20–30 km from the hospital (18). In Kenya, a differently themed study established that admission rates decreased by 11–20% per 5-km increase in distance from a care facility (19). In Figure 2, we demonstrated a strong bias in the distribution of health facilities particularly in regions bordering Sudan, Ethiopia, and Djibouti. It should be noted that all these countries have suboptimal MCV coverage – Ethiopia [MCV1 = 59% and MCV2 9% in 2019 (20); Sudan (MCV1 = 90% and MCV2 = 72% in 2017) (20), and Djibouti (MCV1 = 72% and MCV2 = 82% in 2016) (15)]. Collectively, the true incidence of measles in hard-to-reach communities that border these countries is hard to gauge.

As progress is made toward elimination of measles, knowledge of age distribution patterns in local populations can be crucial for targeted MCV campaigns (16). In this analysis, the age-based analysis of CIR and ASR revealed that measles cases were higher in adolescents and adults. Among the 608 (21.9%) laboratory confirmed cases, 53 (9.92%) were < 1-year-old. This finding corroborates previous reports that suggested that the median age for measles susceptibility is high and that measles incidence is largely driven by susceptible adolescents and adults (8). According to Prada and colleagues, the achievement of the required MCV thresholds can have a dual impact on the epidemiology of measles – it can lengthen the inter-epidemic period and increase the average / or median age at infection (21). The epidemiological shift to older cohorts is therefore an indicator of progress. From a policy perspective, the data suggests that SIA targeting children from 9 months to 14 years of age (as was the case in 2003–2018) (8) will not reach a significant number of susceptible individuals.

The scaling of the measles distribution with age has been linked to multiple factors. First, the distribution of measles can be influenced by birth rates and overall age structure (16). Obviously, high birth rates will skew incidence towards younger cohorts – the converse also applies. Furthermore, we must note the fact that annual vaccine coverage reports in WHO repositories do not involve all age groups. The focus of these reports is mostly restricted to infants. Consequently, WHO country reports often ignore the possible ways in which age-related factors, such as suboptimal vaccination in previous years (say 20 years ago), vaccine failure (primary or secondary) (22), and temporal variations in vaccine effectiveness or quality, can undermine herd immunity thresholds (23). In Eritrea, for example, MCV1 coverage in 1993 was 34% and sustained MCV1 coverage >95% was achieved in 2003 (8). Importantly, routine coverage of MCV2 was initiated in 2012 and required thresholds (>85%) have not been met. Based on these reports, we can conclude that a significant number of young adults and adolescents were either unvaccinated or under vaccinated. Therefore, the increased susceptibility to measles in adolescents and young adults observed in this setting aligns with expectation. This determination is broadly supported by the result showing an 89% decrease in the likelihood of being vaccinated per year of age increase.

Another concerning issue that should be highlighted is the possibility that immunity levels of the population may not be as high as the coverage data suggest. This was recently demonstrated by a study carried out on a small population of Eritreans abroad (average age: 25 years). In this study, the researchers demonstrated that only 76.4% of the study participants had the required concentration of protective antibody titers for measles (24). A different study placed the figure at a marginally higher proportion, 79.9% (25). In all, our data suggest (at least partly) that residual susceptibility inherited from the underperformance of vaccination campaigns (MCV2 in particular) (26) in the past decades continues to undermine measles elimination efforts. Therefore, the need for serological surveys (a direct measure of immunity) and more frequent catch-up and follow-up campaigns targeting unvaccinated adolescents and young adults living in rural, hard-to-reach communities is warranted.

Further stand-out features of our results include the low mortality rates, potential improvements in case notification efficiency, and as previously noted (8); gaps in surveillance performance, particularly, laboratory analysis of suspected cases and epidemiological investigations. In general, much of the discussion on the short-comings of passive surveillance applies to data on measles case fatality rates (CFR). Regarding measles surveillance, we noted a significant under-performance in the laboratory investigation of suspected cases of measles in recent years. The spatial analysis demonstrated a disjunction between regions with higher test rates and regions with measles induced rash. In other words, regions with high prevalence of measles-like symptoms were under-tested. For example, from 2017 to 2020, suspected cases from subzones bordering regions (Zone 1 and Zone 2 in the Afar region in Ethiopia) with some of the lowest coverage of MCV1 and MCV2 vaccine coverage (~ 29.6%) (27, 28) were not tested. Similarly, regions neighboring Sudan or Djibouti are either under-tested or not tested. Altogether, our results are in line with a previous study in Eritrea which indicated that the country has not attained the 80% target for reporting suspected measles cases with blood specimen since 2011 (8). Other investigators in the region have reported similar degeneration in measles surveillance (29).

According to the WHO, suspected measles cases should be confirmed in the laboratory using serological tests or Nucleic Acid based tests (RT-PCR). This requirement is based on the assumption that the positive predictive value (PPV) of clinical case definition is low in settings with a low incidence of measles. The problem of low PPV can be compounded by the high prevalence of viruses that can cause morbiliform rashes (30). As noted earlier, the official WHO-UNICEF or WHO Eritrea Biennium Report 2020–2021 (31) suggest that the incidence of measles in Eritrea is low and the prevalence of viruses that can cause measles-like rashes is growing; therefore, comprehensive case investigation, documentation, and reporting is essential. Another allied concern was the observed discrepancy in the number of suspected cases of measles documented in IDSR and the numbers reported in the official WHO-UNICEF Joint Reporting Form (JRF). As noted previously, the discrepancy points to gaps in reporting and the need for data harmonization.

In a separate analysis, we investigated the epidemiology of laboratory confirmed measles cases. According to our analysis, the diagnosis of measles was associated with age, address, facility type, vaccination status, and year of the onset of rash. In the logistic regression analysis, we showed that children <5–9 years had a lower likelihood of measles-like rash compared to children <4 years. This finding is supported by previous research (16) and has been linked to the age of eligibility for MCV. In general, MCV1 is given at 9 months in Eritrea. Therefore, a significant number of children <1 years are unvaccinated and are thus susceptible to measles. As noted previously, the significant age-dependent increase in the likelihood of measles-like rash in individuals >10 years is probably related to underperformance of vaccination campaigns in the past.

Similar to other studies in the region (32), we observed a significant geographical variation in the likelihood of test positivity. Predictably, the likelihood of test positivity was lower in Zoba Maekel; the zone where testing is concentrated and vaccine coverage is higher. Regions bordering Sudan (Gash-Barka and NRS) and Ethiopia and Djibouti (SRS) had the highest likelihood of test positivity (and low testing density as noted earlier). Remarkably, SRS which borders regions in Ethiopia with extremely low vaccination coverage (Afar region – Zone 1 and Zone 2 have vaccine coverage <30%) (27, 28); had the highest likelihood of measles test positivity. Furthermore, NRS and Gash-Barka, which borders administrative regions in Sudan [Red Sea and Kasala – the two regions have relatively frequent measles outbreaks (33)]; had a high number of patients with measles-like rash, high test positivity, and low number of tests. Compared to Maekel, the likelihood of being unvaccinated in these regions was relatively high. A similar finding was reported in a previous study (26). Previously, we suggested that parts of NRS, Gash Barka, and SRS have relatively low density of health infrastructure and that this can lead to spatial inequalities in routine vaccine coverage or testing. Another factor that can undermine vaccine coverage in these settings is the fact that some communities living in these regions have nomadic and semi-nomadic lifestyle.

Although our analysis has shown a higher measles positivity rate in patients with measles-like rash in NRS, Gash Barka, SRS, and the presence of a host of factors that can undermine measles vaccine coverage, testing or reporting; we are not suggesting that measles may be endemic in these regions. Previous mathematical calculations addressing the epidemiology of measles have shown that a population of 250,000–500,000 people is required to establish measles as an endemic disease (16). In contrast, NRS and SRS are sparsely populated and settlements are relatively isolated, suggesting that measles outbreaks are largely due to persistent reintroduction from other areas. Furthermore, social contact clusters in these regions often cut across national borders. This concern was recently reiterated in a WHO report suggesting that despite high measles vaccines in Eritrea, the country is at risk of measles importation due to cross-border population movement and potential population mix dynamics (31). Additional support for this conclusion is related to the fact that the impact of low vaccination coverage and the endemicity of measles in surrounding countries is known to be more pronounced in smaller countries like Eritrea (16). However, in the absence of realistic data on social mixing patterns in cross-border communities and genotypic data; tracking of the sources of outbreaks or transmission chains in the country is difficult. In summary, we conclude that even if the conditions for the elimination of measles elimination (MCV1 > 95% and MCV2 > 85% in all regions) are met in Eritrea; sustained elimination will be difficult due to the high odds of transmission across the border from highly impacted, under-vaccinated sub-regions in Sudan and Ethiopia. Therefore, the importance of regional coordination of elimination efforts and robust surveillance in NRS, SRS, and Gash-Barka cannot be overemphasized.

Last but not least, we showed that patients in hospitals had a higher probability of test positivity compared to patients from health centers/stations. A plausible explanation for this finding relates to the possibility that patients with measles-related complications are usually referred by primary health clinics (PHC) to hospitals where trained physicians are stationed. Unlike caregivers in PHCs; physicians in hospitals may be better at diagnosis and profiling. Separately, we found a connection between measles test positivity and vaccination. This outcome is supported by previous studies done in Ethiopia (18, 34), South Sudan (35), and Gambia (32). Interestingly, individuals who were vaccinated before 2010 had a higher likelihood of measles test positivity. At present, we have no obvious explanation for this outcome. However, we believe that the proximity between this timeline and the initiation of MCV2 coverage in 2012 is not coincidental.

### Strength and limitation

Unlike previous work which utilized data from WHO/UNICEF Joint Reporting Form (JRF) and administrative data, we sourced our data from the national and sub-national representative DHS database. This notwithstanding, our study suffers from a number of limitations including inconsistencies in measles testing between sub regions and over time. Additionally, information on vaccination status was based, to a large extent, on self-reported responses. Therefore, social desirability bias cannot be discounted. Last but not least, retrospective studies typically suffer from missing data problems, and we believe that this study was not an exception.

## Conclusion

Our report suggests that Eritrea has a relatively low incidence of measles but a high variability year-to-year. However, the potential for under-estimation of cases exists due to under-reporting of suspected cases and incomplete testing, particularly in recent years. Furthermore, age-based analysis of CIR and ASR revealed that the median age for measles cases was 7 years (IQR: 4–14 years). This means that SIV campaigns targeting individuals <14 years will miss a large proportion of individuals with residual susceptibility. Laboratory positivity for measles was associated with increasing age, residence in border regions (Gash-Barka, SRS, and NRS), level of care facility, vaccination status, and year of onset of measles-like rash. Interestingly, uptake of MCV was associated with a similar complement of factors. In a separate analysis, we demonstrated that despite the high coverage of MCV in Eritrea, spatial inequalities in vaccine coverage or surveillance and extremely low vaccination rates in specific sub-regions in neighboring countries continue to undermine elimination efforts. To address these issues, there should be more focus on regional micro planning to create efficient systems for monitoring, reporting, and management of outbreaks. Additional strategies can include decentralization of testing; targeted serological surveys; ring vaccination in border areas; more frequent catch-up SIAs targeting unvaccinated adults and adolescents; building capacity for genetic testing of isolates, and enhancing regional coordination of elimination efforts.

## Data availability statement

The raw data supporting the conclusions of this article will be made available by the authors, without undue reservation.

## Ethics statement

The requirement of ethical approval was waived by the study was carried out according to the Declaration of Helsinki and was approved by the Ethical Committee of the Eritrean Ministry of Health (EMH). Written consent was not obtained from the patients. Instead, the data were identified and all ethical and professional considerations undergirding confidentiality/or other protections of patient information were rigorously implemented. For the studies on humans because written consent was not obtained from the patients. Instead, the data were identified and all ethical and professional considerations undergirding confidentiality/or other protections of patient information were rigorously implemented. The studies were conducted in accordance with the local legislation and institutional requirements. Written informed consent for participation was not required from the participants or the participants’ legal guardians/next of kin in accordance with the national legislation and institutional requirements. The human samples used in this study were acquired from a by- product of routine care or industry.

## Author contributions

SM conceived and designed the study. SM, AT, FT, IM, MS, DF, HA, RM, TT, and LW participated in the performed data collection. SM and OA did the statistical analysis and spatial mapping. SM, OA, MH, and WW did the data presentation and reviewed and edited the manuscript. All authors read and approved the final manuscript.

## Conflict of interest

The authors declare that the research was conducted in the absence of any commercial or financial relationships that could be construed as a potential conflict of interest.

## Publisher’s note

All claims expressed in this article are solely those of the authors and do not necessarily represent those of their affiliated organizations, or those of the publisher, the editors and the reviewers. Any product that may be evaluated in this article, or claim that may be made by its manufacturer, is not guaranteed or endorsed by the publisher.
